# Genome-wide identification of *SWEET* genes reveals their roles during seed development in peanuts

**DOI:** 10.1186/s12864-024-10173-w

**Published:** 2024-03-07

**Authors:** Yang Li, Mengjia Fu, Jiaming Li, Jie Wu, Zhenyang Shua, Tiantian Chen, Wen Yao, Dongxin Huai

**Affiliations:** 1 College of Life Sciences, Henan Agricultural University, 450046 Zhengzhou, China; 2 Key Laboratory of Biology and Genetic Improvement of Oil Crops, Ministry of Agriculture and Rural Affairs, Oil Crops Research Institute of Chinese Academy of Agricultural Sciences, Wuhan, China

**Keywords:** Peanut, SWEET, Gene family, Seed development, Expression analysis

## Abstract

Sugar Will Eventually be Exported Transporter (SWEET) proteins are highly conserved in various organisms and play crucial roles in sugar transport processes. However, SWEET proteins in peanuts, an essential leguminous crop worldwide, remain lacking in systematic characterization. Here, we identified 94 *SWEET* genes encoding the conservative MtN3/saliva domains in three peanut species, including 47 in *Arachis hypogea*, 23 in *Arachis duranensis*, and 24 in *Arachis ipaensis*. We observed significant variations in the exon-intron structure of these genes, while the motifs and domain structures remained highly conserved. Phylogenetic analysis enabled us to categorize the predicted 286 SWEET proteins from eleven species into seven distinct groups. Whole genome duplication/segment duplication and tandem duplication were the primary mechanisms contributing to the expansion of the total number of *SWEET* genes. In addition, an investigation of *cis*-elements in the potential promoter regions and expression profiles across 22 samples uncovered the diverse expression patterns of *AhSWEET* genes in peanuts. *AhSWEET24*, with the highest expression level in seeds from *A. hypogaea* Tifrunner, was observed to be localized on both the plasma membrane and endoplasmic reticulum membrane. Moreover, qRT-PCR results suggested that twelve seed-expressed *AhSWEET* genes were important in the regulation of seed development across four different peanut varieties. Together, our results provide a foundational basis for future investigations into the functions of *SWEET* genes in peanuts, especially in the process of seed development.

**Supplementary Information:**

The online version contains supplementary material available at 10.1186/s12864-024-10173-w.

## Background

Currently, three distinct superfamilies of sugar transporters have been identified in plants, which include sucrose transporters (SUTs), monosaccharide transporters (MSTs), and sugar will eventually be exported transporter (SWEET) proteins [1, 2]. SWEET proteins, which function as sugar uniporters, exhibit a high degree of conservation and are widely distributed in plants, animals, fungi, bacteria, and archaea [3]. The first identification of *MtN3*, a member of the SWEET family, revealed its role in root nodule development in *Medicago truncatula* [4]. In 2010, this class of sugar transporters was originally designated as SWEET [5]. Plant SWEETs are characterized by seven transmembrane helixes (TMs) that encompass two internal triple-helix bundles (THBs) or two MtN3/saliva domains [5–7]. The cryo-EM structure of OsSWEET2b from *Oryza sativa* showed that N-terminal and C-terminal THBs comprised TM1-TM3-TM2 and TM5-TM7-TM6, respectively [6]. The TM4 served as an intervening linker, connecting the two THBs, or MtN3/saliva domains. Therefore, the structural configuration of plant SWEETs is described as having a 3 + 1 + 3 topology. However, SemiSWEETs in prokaryotes and SuperSWEETs in oomycetes possess three and over eighteen TMs, respectively [8, 9].

As sugar transporters, SWEETs in plants mediate the transport of hexoses, sucrose, and fructose [10–12]. Accumulating studies have demonstrated the significant roles played by SWEETs in various aspects of plant growth and development, such as root, leaf, flower, and seed development, as well as responses to biotic and abiotic stresses. In *Arabidopsis*, it was observed that *AtSWEET17*, dependent on fructose, had an impact on root development under drought stress [13]. Additionally, *AtSWEET11* and *AtSWEET12* were identified as regulators of sucrose efflux in leaf phloem [10]. The overexpression of *AtSWEET10* in *Arabidopsis thaliana* was shown to affect flowering under long-day conditions [14]. A triple mutant (*atsweet11;12;15*) exhibited a “wrinkled” seed phenotype, characterized by delayed embryo development, as well as decreased seed weight, starch content and lipid content [15]. In rice, the suppression of *Os8N3* was linked to enhanced resistance against the *Xanthomonas oryzae* pv. *Oryzae* strain PXO99^A^ which can cause bacterial blight in rice [16]. Moreover, *OsSWEET13* and *OsSWEET15* were found to impact salinity and drought tolerance through the ABA-signaling pathway [17].

The cultivated peanut (*Arachis hypogaea* L.) stands as one of the world’s most important crops and provides valuable sources of seed oil and protein for human consumption (https://www.fao.org/). Over the years, peanut breeding and genetic improvement have primarily revolved around the objectives of achieving higher yields and enhancing oil and protein qualities. Although several genes associated with peanut seed development, such as *AhRUVBL2* and *PSW1*, have been successfully identified, the intricate molecular mechanisms underlying this process largely remain elusive [18, 19]. The availability of multiple peanut genomes and transcriptomes provide an invaluable resource for identifying potential candidate genes associated with peanut seed development [20–24]. In this study, a comprehensive genome-wide investigation and analysis of *SWEET* gene family were conducted in a cultivated peanut *A. hypogea* and its two diploid ancestral species *A*. *duranensis* and *A*. *ipaensis*. A total of 94 *SWEET* genes were identified, distributed across diverse chromosomes. Moreover, gene structure, conserved motif, phylogenetic relationships, collinearity, *cis*-elements of promoter region, expression patterns and subcellular localization were analyzed and explored. Our findings unveiled the association of certain peanut *SWEET* genes with seed development.

## Results

### Characterization of SWEET family members in *Arachis* spp

A total of 94 potential SWEET proteins in three peanut species were found, with 47 in *A. hypogea* (AABB genome), 23 in *A. duranensis* (AA genome), and 24 in *A. ipaensis* (BB genome) (Table S1). Compared to the two model plants, *O*. *sativa* (21 SWEETs) and *A*. *thaliana* (17 SWEETs), it became evident that the number of SWEET proteins was notably higher in *A. hypogea* [5, 7]. In addition, the coding sequences (CDSs) of peanut *SWEET* genes ranged from 390 to 1,035 bp in length, encoding proteins consisting of 129 to 344 amino acids, with molecular weights from 15.01 to 38.84 kDa (Fig. S1; Table S1). Moreover, the results of predicted subcellular localization showed that all 94 SWEET proteins might be localized in the plasma membrane, which was consistent with earlier results for other species [2, 5, 25].

Subsequently, the chromosomal distributions of 94 *SWEET* genes in peanuts were investigated. In *A. hypogaea*, *AhSWEET* genes were mapped to Chromosomes 03–08, 10 and 13–20 (Fig. S2). Notably, Chromosome 03 possessed the highest number of *AhSWEET* genes with seven members, closely followed by Chromosome 04 and 13, each containing five members. In contrast, Chromosome 07, 10 and 19 contained the lowest numbers of *AhSWEET* genes, with only one member for each. In *A. duranensis*, 60.87% of *AdSWEET* genes were distributed on the A03, A04 and A08 chromosomes. There were no *AdSWEET* genes detected on Chromosome A02 and A09. In *A. ipaensis*, seven out of 24 *AiSWEET* genes were localized to Chromosome B03, while individual members were situated on Chromosome B01, B09 and B10. These findings illustrated that *SWEET* genes were widely but unevenly distributed on peanut chromosomes.

We further identified 26 orthologous groups of *SWEET* genes in *A. hypogea* and its wild ancestors (*A. duranensis* and *A. ipaensis*) (Table S2). It was observed that 20 (86.96%) *AdSWEETs* and 20 (83.33%) *AiSWEETs* were retained in the allotetraploid peanut species *A. hypogea*, indicating a predominant origin of *SWEET* genes in *A. hypogea* from *A. duranensis* and *A. ipaensis*. *AdSWEET20* and *AdSWEET23* were not found or were lost in the other two genomes. *AiSWEET7* and *AiSWEET8*, as well as *AhSWEET1* and *AhSWEET9* exhibited the same outcomes. It suggested that these genes might be species-specific *SWEETs*.

### Conservation and divergence of gene structures, domains, and motifs of peanut *SWEET* genes

**Fig. 1:**
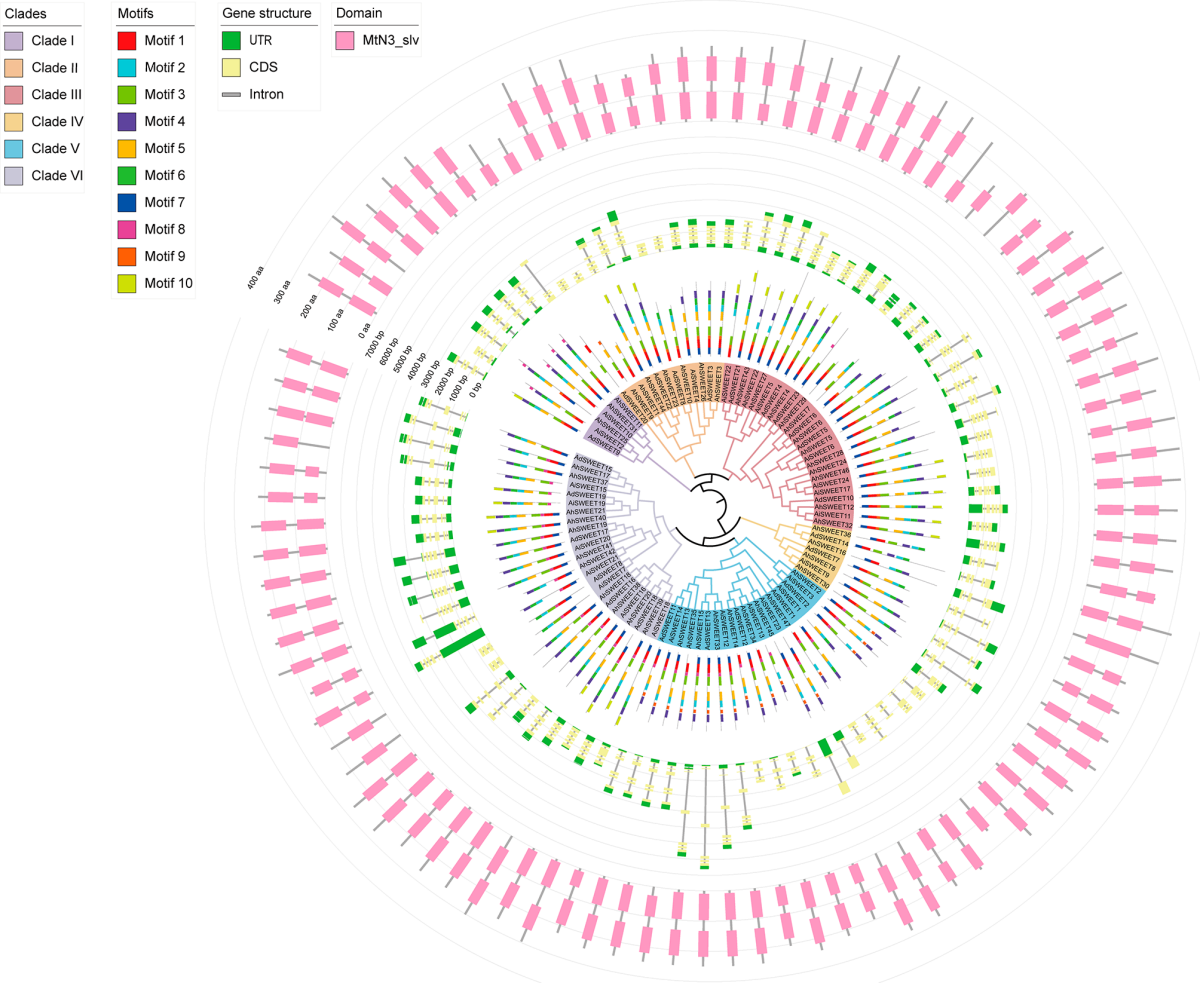
Conserved domains, gene structure, motif, and phylogenetic relationship of the 94 *SWEET* genes among three peanut species. Tracks, from inside to outside, represent the phylogenetic relationship, motif, exon-intron structure, and domain structure

To gain insights into the diversity in gene structures among peanut *SWEET* genes, the exon-intron organizations were analyzed based on genomic sequences. Our findings demonstrated a wide variation in the number of exons and introns among *SWEET* genes in the three peanut species, ranged from two to eight exons and one to seven introns (Fig. 1, Fig. S1, and Table S1). It is noteworthy that a significant proportion of peanut *SWEET* genes exhibited six exons (41 out of 94, accounting for 43.62%) and seven exons (22 out of 94, accounting for 23.40%). The homologous gene pair *AhSWEET45* and *AiSWEET23* possessed the lowest exon number, with only two, while *AhSWEET41* and *AiSWEET20* exhibited the highest number, with eight exons. Additionally, based on phylogenetic analysis, peanut *SWEET* genes within the same clade exhibited gene structural similarities.

The conserved MtN3/saliva domain represents a canonical domain for SWEET proteins. In general, a SWEET protein is composed of seven *α*-helical transmembrane domains, encompassing two MtN3/saliva domain [7]. SMART analysis revealed that all peanut SWEET proteins possessed four to eight TM helices (Table S1). Additionality, among the 94 SWEET proteins, the majority (~ 89.36%) featured two MtN3/saliva domains, while ten SWEETs shared a single MtN3/saliva domain (Fig. 1), indicated the functional diversity of SWEET proteins in peanuts.

We next investigated the conserved motifs in peanut SWEET proteins using the MEME tool (Fig. 1 and Table S3). Our analysis observed that approximately 92% of peanut SWEET proteins shared common motifs, including motif1 (87 out of 94), motif2 (86 out of 94) and motif3 (86 out of 94) (Fig. 1). However, motif10 was identified in only 26 members. A total of nine distinct motifs were found in 24.47% of all SWEET proteins. Notably, AdSWEET20 exhibited the lowest number of motifs, consisting of only motif1 and motif3. Furthermore, the examination of motifs and the phylogeny of peanut *SWEET* genes revealed the widespread distribution of seven motifs (motif1 to motif7) across six clades. However, three motifs (motif8 to motif10) were found to be restricted to specific clades. Motif8 was absent in Clade II and Clade IV, motif9 was not identified in Clade VI, and motif10 was not detected in Clade IV and Clade V.

We also observed that homologous *SWEET* genes in different peanut species exhibited close phylogenetic relationships and gene characteristics, such as gene structure, domain and motif. For instance, the orthologous gene pairs (e.g., *AhSWEET3*/*AdSWEET3*, *AhSWEET32*/*AiSWEET11*) and paralogous gene pairs (e.g., *AhSWEET11*/*AhSWEET31*, *AiSWEET7*/*AiSWEET8*) revealed a high degree of similarity in exon-intron structure and conservation of domains and motifs. The above findings indicated both structural conservation and divergence among *SWEET* genes in peanuts.

### Phylogenetic relationship of *SWEET* genes

**Fig. 2:**
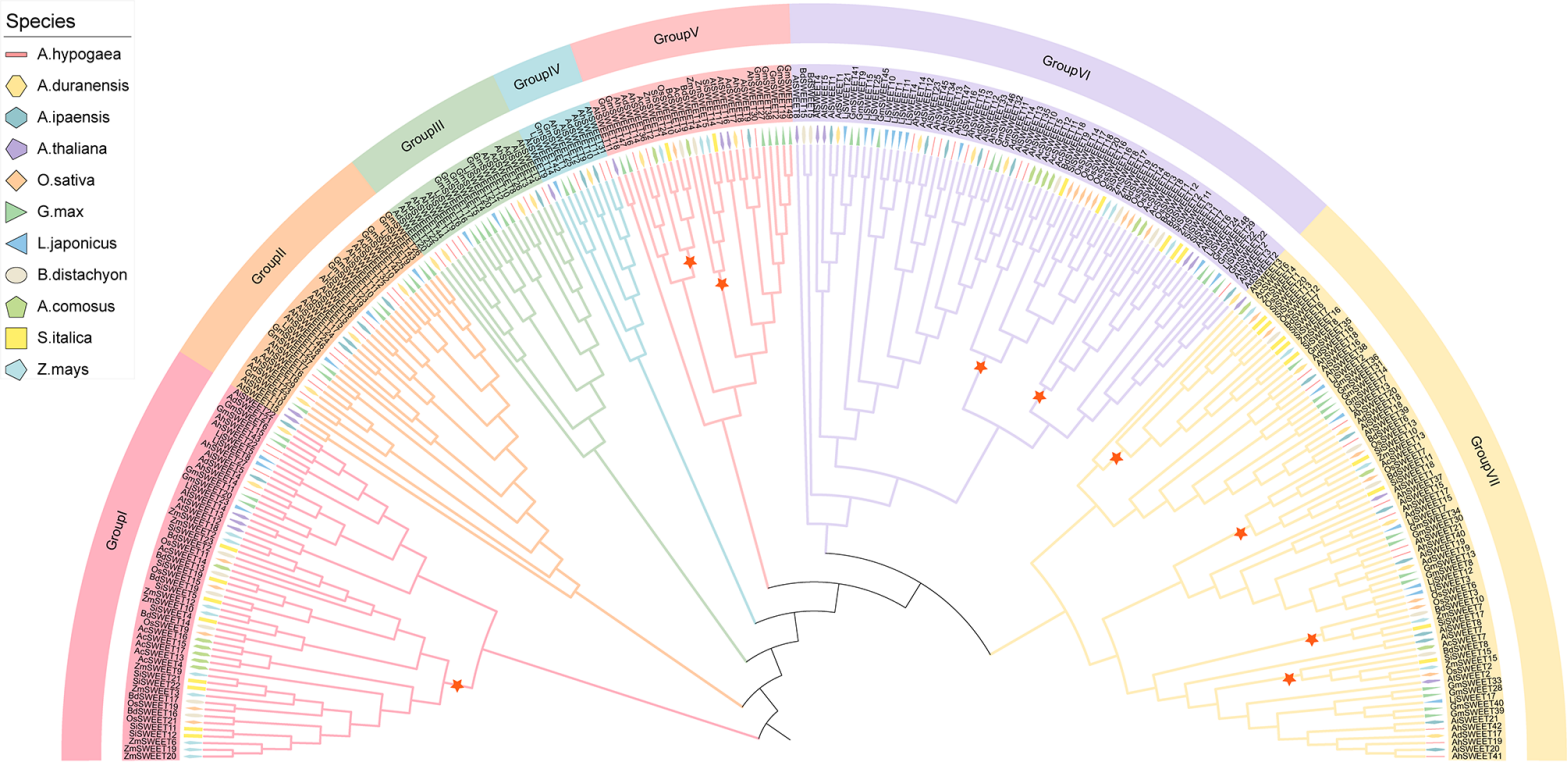
Phylogenetic relationships of 286 SWEET proteins from eleven species. The SWEET family members were categorized into seven groups, each represented by a unique distinct color. Various shapes denoted different plant species (*O. sativa*, *B*. *distachyon*, *S*. *italica*, *Z*. *mays*, *A*. *comosus*, *A. thaliana*, *A. hypogaea*, *A. duranensis*, *A. ipaensis*, *G. max*, and *L. japonicus*). The clades composed of monocot *SWEET* genes are highlighted by red stars

To evaluate the evolutionary relationships among SWEET proteins in peanuts and other species, a phylogenetic tree was constructed through a multiple sequence alignment using predicted SWEET protein sequences. These sequences were sourced from various monocots: *O. sativa* (21 SWEETs), *Brachypodium distachyon* (19 SWEETs), *Setaria italica* (24 SWEETs), *Zea mays* (20 SWEETs), and *Ananas comosus* (17 SWEETs), as well as from the eudicots: *A. thaliana* (17 SWEETs), *A. hypogaea* (47 SWEETs), *A. duranensis* (23 SWEETs), *A. ipaensis* (24 SWEETs), *Glycine max* (49 SWEETs), and *Lotus japonicus* (25 SWEETs) (Table S4). In total, the 286 SWEET proteins were divided into seven distinct groups (Fig. 2). Among these groups, SWEET proteins from eudicots and monocots were distributed across four of the seven groups (Group I, V, VI, and VII), while Group II, III and IV exclusively contained eudicot species (*A. thaliana*, three peanut species, *G. max*, or *L. japonicus*). In Groups I, V, VI and VII, monocot SWEETs were clustered together, indicating close phylogenetic relationships and a high level of conservation (Fig. 2).

SWEET proteins from the three peanut species were distributed across all seven groups. Group VII contained the largest number of peanut SWEETs (24 members), while Group IV had the fewest, with only six members. Moreover, we found that peanut SWEETs exhibited a closer relationship with legume SWEET proteins (*G. max* and *L. japonicus*), compared to those from five monocots and *A*. *thaliana*. Group III included only the SWEET proteins from five leguminous plants, indicating functional conservation among these members. Consequently, SWEET members from closely related species tended to be grouped together.

### Collinearity and purifying selection of *SWEETs* in peanuts

**Fig. 3:**
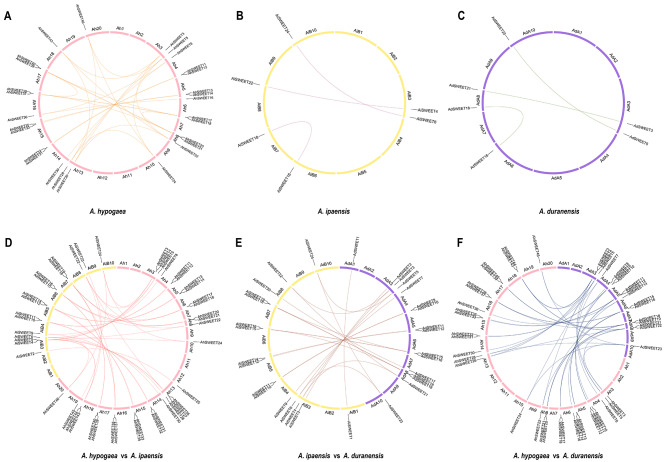
Collinearity analysis of *SWEET* genes in peanuts. Collinear gene pairs of *SWEETs* in *A. hypogaea* (**A**), *A. duranensis* (**B**), and *A. ipaensis* (**C**). Collinear gene pairs of *SWEETs* between *A. hypogaea* and *A. ipaensis* (**D**). Collinear gene pairs of *SWEETs* between *A. ipaensis* and *A. duranensis* (**E**). Collinear gene pairs of *SWEETs* between *A. hypogaea* and *A. duranensis* (**F**). Different-color lines highlight the WGD/segment duplicated gene pairs

To gain a deeper understanding of the phylogenetic mechanisms occurring in the peanut SWEET family, we investigated the duplication events, including WGD/segment duplication and tandem duplication, across three peanut species. A total of 73 *SWEET* genes from three peanut genomes were found to anchor to syntenic blocks. Through intragenomic comparison analysis, a total of 27 collinear gene pairs in *A. hypogaea* were obtained (Fig. 3A and Table S5). It is worth noting that some genes were involved in multiple gene pairs, such as *AhSWEET5*/*AhSWEET24*, *AhSWET5*/*AhSWEET28*, and *AhSWEET5*/*AhSWEET46*. In *A. duranensis*, three collinear gene pairs were found, consistent with *A. ipaensis*, which also had three collinear gene pairs (Fig. 3B and C, Table S5). Upon conducting intergenomic comparisons, there were 41 gene pairs between *A. hypogaea* and *A. ipaensis*, 42 gene pairs between *A. hypogaea* and *A. duranensis*, and 22 gene pairs between *A. ipaensis* and *A. duranensis* (Fig. 3D, E and F, and Table S5). Significantly, ~ 56.52% of all *SWEET* collinear gene pairs were located in highly conserved syntenic blocks that possessed more than 200 genes.

Additionally, a total of nine, three and four tandem duplication genes in *A. hypogaea*, *A. duranensis* and *A. ipaensis* were identified, respectively (Table S6). Among these, three tandem duplication genes, including *AhSWEET41*-*AhSWEET42*, *AiSWEET7*-*AiSWEET8*, and *AiSWEET20*-*AiSWEET21*, were also found to be homologous gene pairs. Notably, the number of WGDs/segment duplications, totaling 138 collinear gene pairs in both the intragenomic and intergenomic comparisons, exceeded that of tandem duplications (16), highlighting the dominant role played by WGDs/segment duplications in the expansion of *SWEET* genes in the three peanut species. Nevertheless, it is important to acknowledge that tandem duplication also served as an essential driving force during the expansion of the *SWEET* gene family.

To assess the selection pressures, we conducted an analysis to calculate the nonsynonymous (Ka) and synonymous (Ks) substitution rates for each duplicated *SWEET* gene pair from the three peanut species (Fig. S3 and Table S7). As a result, the Ka/Ks ratios for the remaining duplicated gene pairs were found to be less than 1, with the exception of *AhSWEET17*-*AhSWEET37*, *AhSWEET5*-*AdSWEET6*, *AhSWEET10*-*AdSWEET9*, *AiSWEET4*-*AhSWEET26*, *AiSWEET6*-*AhSWEET28*, and *AiSWEET22*-*AhSWEET43*. We also observed that all duplicated *SWEET* gene pairs in the two diploid peanut species, as well as between *A. duranensis* and *A. ipaensis*, had Ka/Ks values < 1. These results showed that the majority of *SWEET* genes were subject to purifying selection, thereby reflecting a high degree of conservation in the peanut *SWEET* gene family during evolution.

### *Cis*-elements analysis of *SWEET* genes in peanuts

**Fig. 4:**
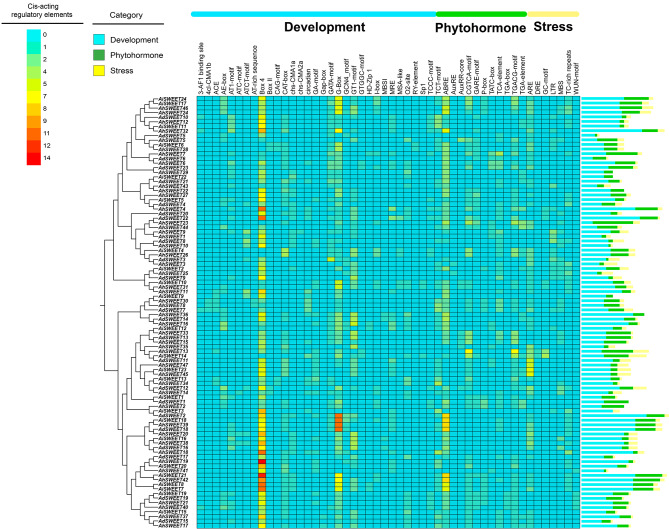
Predicted *cis*-elements in the promoter regions of *SWEET* genes in three peanut species. Three distinct groups of *cis*-elements are displayed, including development, phytohormone, and stress. The vertical axis indicates the number of 94 *SWEET* genes in each *cis*-element. The arrangement of *SWEET* genes follows the order of the phylogenetic tree

Promoter plays a crucial role in regulating gene transcription. The *cis*-elements within promoter are essential for determining gene expression level. In tomato, the study have shown that the *cis*-elements of *SWEET* genes were primarily associated with stress responses [26]. To investigate the potential *cis*-elements involved in peanut growth and development, the upstream 2000-bp regions of *SWEET* genes in three peanut species were analyzed using PlantCARE (Fig. 4; Table S8). Abundant *cis*-elements were detected, including thirty-one elements related to development, twelve elements associated with phytohormone responses, and seven elements involved in stress tolerance. Of note, the Box 4 element (ATTAAT, present in 88 *SWEET* genes) and the G-Box element (ACACGTG(G/T)CACC, present in 78 *SWEET* genes), both associated with light responses, were the most numerous *cis*-elements in the potential promoters of peanut *SWEET* genes. Additionally, the ABRE element (CGCACGTGTC), involving in responding to abscisic acid [27], was identified in approximately 77.66% of the 94 *SWEET* genes. The CGTCA-motif (CGTCA), which responds to MeJA, was present in the promoters of 55 *SWEET* genes. The promoters of 66 *SWEET* genes contained ARE element (AAACCA), which were related to anaerobic induction. Moreover, the *cis*-elements identified in the promoters of homologous *SWEET* gene pairs in peanuts exhibited conservation. For instance, an orthologous group comprising *AdSWEET5*, *AiSWEET6*, *AhSWEET5*, and *AhSWEET28* possessed a high number of Box 4 and G-Box elements. These results suggested that the 94 *SWEET* genes might have significant roles in peanut growth and development, as well as in their response to phytohormones and stresses.

### Expression patterns of *SWEET* genes in *A. hypogaea*

**Fig. 5:**
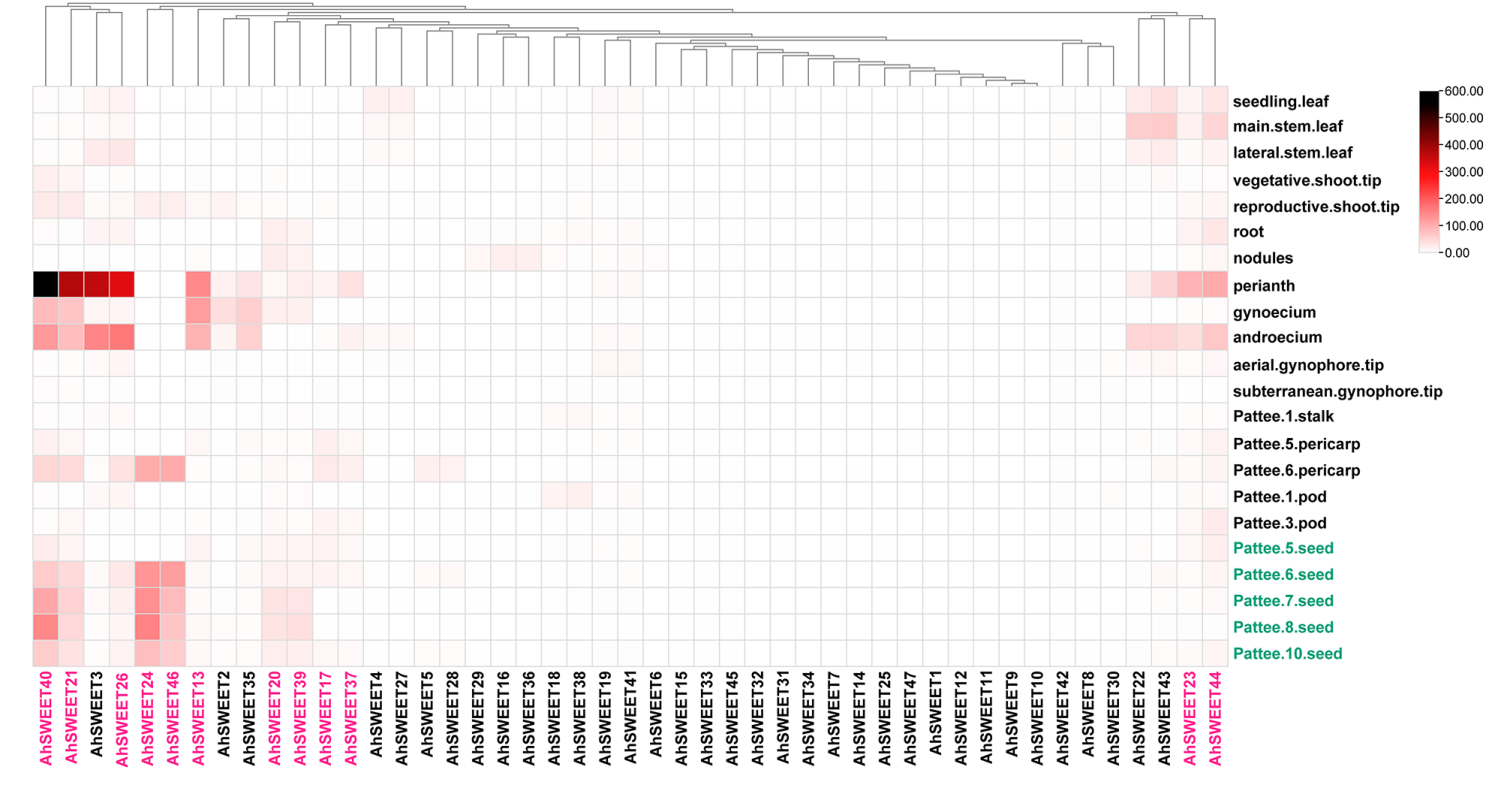
Expression patterns of *AhSWEET* genes in various tissues and stages. The vertical axis indicates the expression patterns of 47 *AhSWEET* genes. The horizontal axis indicates different samples, including leaves (three stages), shoot tips (two stages), root, nodules, perianth, gynoecium, androecium, gynophore tips (two stages), stalk, pericarps (two stages), pods (two stages), and developing seeds (five stages). The red genes indicate twelve *AhSWEET* genes with an FKPM (≥ 10) in any of the five stages of developing seeds (Pattee.5.seed to Pattee.10.seed)

Transcriptome data from different plant tissues and development stages provide valuable insights into the functional divergence of genes. To elucidate the roles of *AhSWEET* genes during different growth and developmental stages in peanuts, the expression patterns of *AhSWEET* genes were analyzed using transcriptome data from the reference *A. hypogaea* Tifrunner [23]. The FPKM values of *AhSWEET* genes across various organs, including roots, shoots, leaves, flowers, pegs, pericarps, seeds, etc., were visually represented in a heatmap figure (Fig. 5). Approximately 72.34% of *AhSWEET* genes (with FPKM ≥ 1) were found to be expressed in at least one tissue, while over a quarter of *AhSWEETs* (13 out of 47) displayed no expression (FPKM < 1) across the various tissues (Table S9). Some *AhSWEET* genes demonstrated high expression levels (FPKM ≥ 30) across three stages in leaf development, perianth, gynoecium, androecium, Pattee.6.pericarp, as well as five stages in seed development. These data highlighted the divergent expression patterns of *AhSWEET* genes, implying their potentially crucial and diverse roles within various peanut tissues and developmental stages.

Interestingly, further investigation showed a relationship between expression pattern and gene homology of *AhSWEET* genes. Most of the homologous gene pairs exhibited highly similar expression trends. For example, *AhSWEET21* and *AhSWEET40* displayed high expression levels in the perianth, gynoecium, androecium, and seed, indicating conservation of functions in regulating peanut development. Surprisingly, *AhSWEET1* and *AhSWEET9*, two *A. hypogaea-*specific genes, showed no expression in any peanut tissues. It is possible that they were pseudogenes or their expressions were required for particular conditions.

It is worth to note that soybean, another significant leguminous oilseed crop, had been reported to express most of the *SWEET* genes during seed development [28]. Based on this, we observed that among the 47 *AhSWEET* genes, twelve members (*AhSWEET13*, *AhSWEET17*, *AhSWEET20*, *AhSWEET21*, *AhSWEET23*, *AhSWEET24*, *AhSWEET26*, *AhSWEET37*, *AhSWEET39*, *AhSWEET40*, *AhSWEET44*, and *AhSWEET46*) exhibited some level of expression in developing peanut seeds (Pattee.5.seed, Pattee.6.seed, Pattee.7.seed, Pattee.8.seed, and Pattee.10.seed), indicating that the twelve genes might have a potential to serve the development of peanut seeds (Fig. 5 and Table S9).

### Subcellular localization of AhSWEET24

**Fig. 6:**
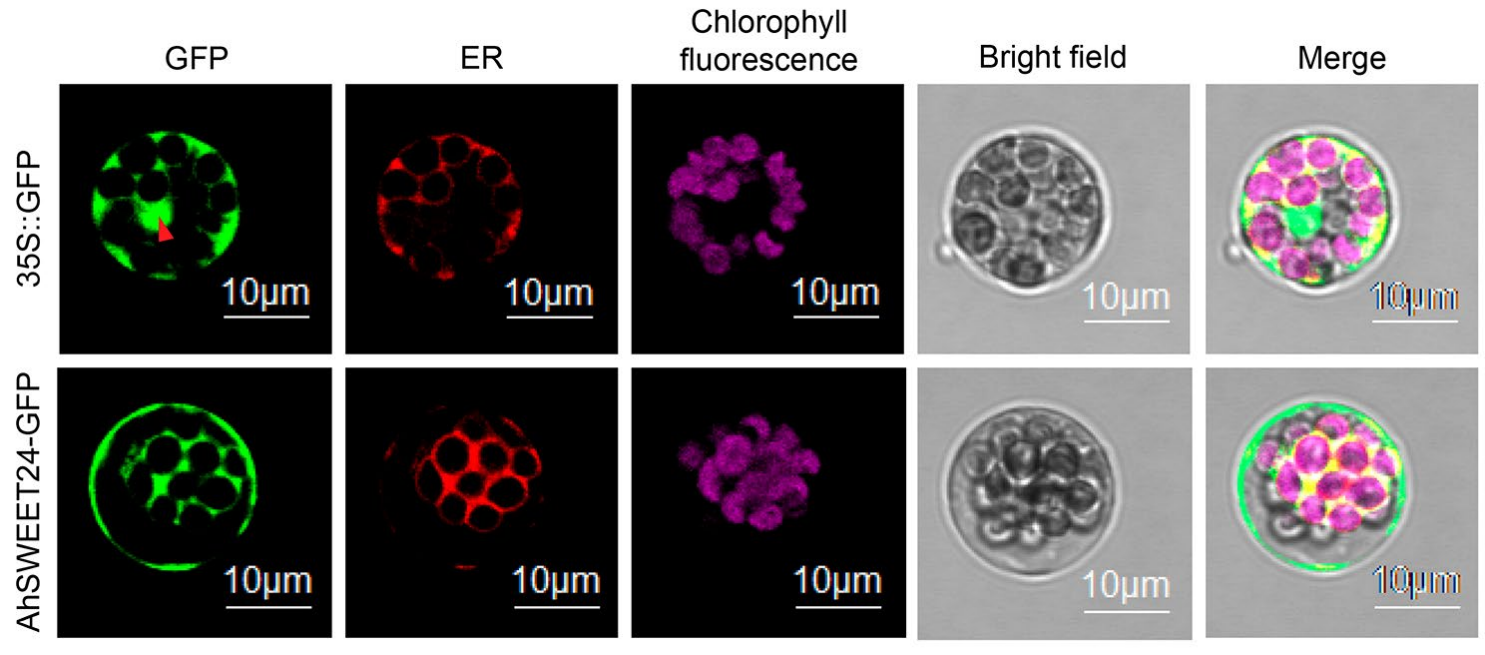
Localization of AhSWEET24 through transient expressions of AhSWEET24-GFP fusion proteins. The 35 S::AhSWEET24-GFP & 35 S::BnaA.FAE1-RFP vectors and 35 S::GFP & 35 S::BnaA.FAE1-RFP vectors were separately co-transfected into *Arabidopsis* protoplasts. Red arrow indicates nucleus

Plant SWEET proteins are membrane-localized proteins, primarily detected in the plasma membrane [29], Golgi membrane [30] and tonoplast [11]. *AhSWEET24* displayed the highest expression levels during four stages of seed development (Pattee 6 seed, Pattee 7 seed, Pattee 8 seed, and Pattee 10 seed) in *A. hypogaea* Tifrunner, suggesting its potential pivotal role in peanut seed development. Consequently, *AhSWEET24* was chosen as a candidate gene for further subcellular localization analysis. The green fluorescence signal of the 35 S::GFP protein was observed throughout the entire cell, including the nucleus, cytoplasm and membranes (Fig. 6). However, the AhSWEET24-GFP fusion protein was existed in the plasma membrane and endoplasmic reticulum membrane. This observation strongly suggested that AhSWEET24 was indeed a membrane-localized protein.

### qRT-PCR validation of *AhSWEETs* during seed development in four peanut varieties

Some *SWEETs* are indispensable for seed development, for example, *AtSWEET11*, *AtSWEET12*, and *AtSWEET15* in *Arabidopsis* [15], as well as *OsSWEET11* and *OsSWEET15* in rice [31]. However, there has been limited research focused on exploring the relationship between *SWEETs* and seed development in peanuts. The transcriptome data from *A. hypogaea* Tifrunner revealed that twelve out of forty-seven *AhSWEET* genes were expressed in developing peanut seeds (Fig. 5). Hence, to further explore the potential functions of the twelve *AhSWEET* genes during seed development, we selected four peanut varieties, including Nanyangbaipi (NYBP), Zhonghua24 (ZH24), SY131, and Jihuatian1 (JHT1). The expression characteristics of the twelve *AhSWEET* genes across five stages of seed development [20 days after pollination (DAP), 30 DAP, 40 DAP, 50 DAP, and 60 DAP] were analyzed using qRT-PCR.

**Fig. 7:**
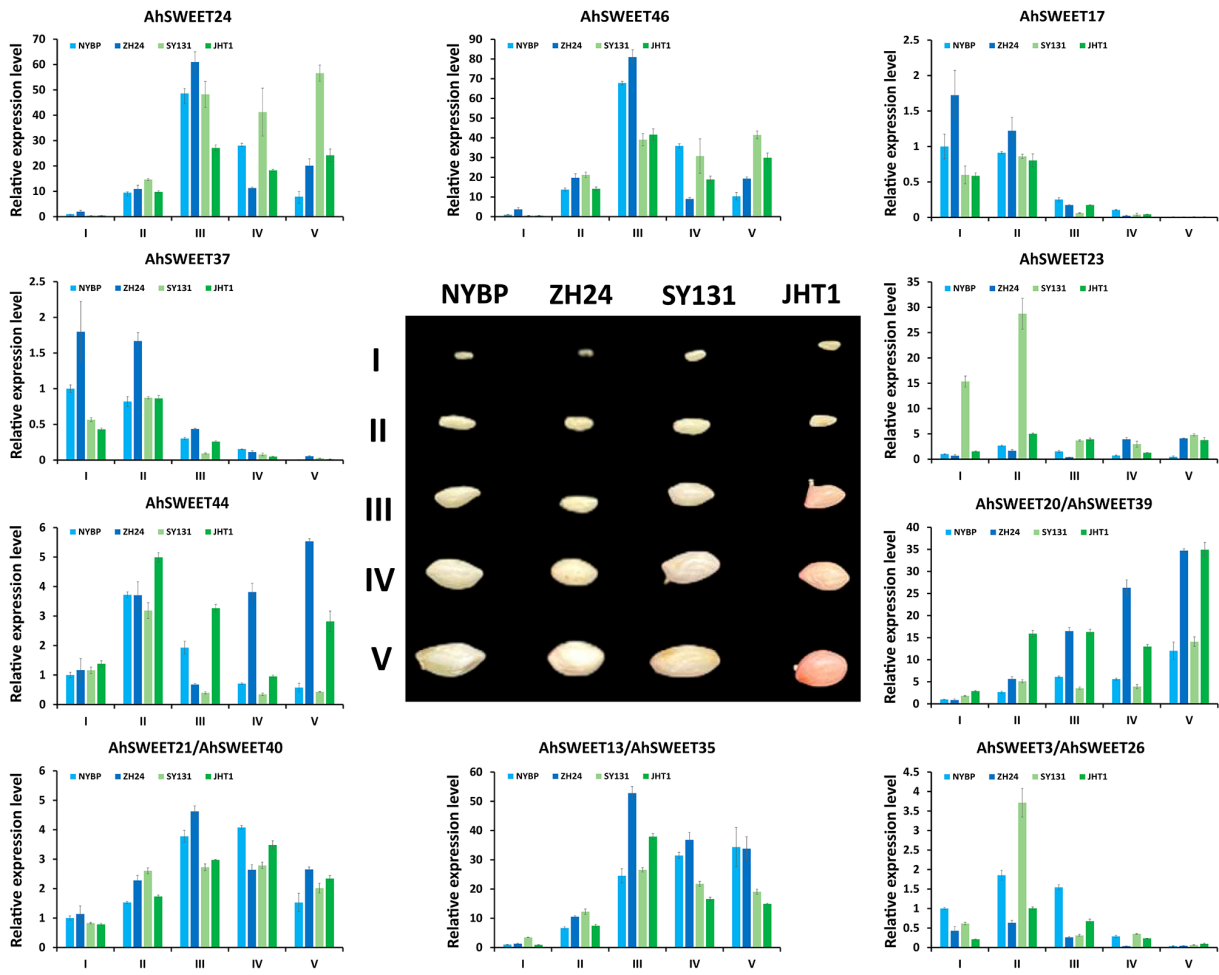
qRT-PCR analysis of twelve *AhSWEETs* expressions at five stages of seed development across four peanut varieties. The five stages of seed development, including 20 DAP, 30 DAP, 40 DAP, 50 DAP and 60 DAP, are denoted as I, II, III, IV and V, respectively. *AhACTIN* served as the internal control. Error bars represent the standard deviation calculated from three biological replicates

The results exhibited various expression patterns among the twelve *AhSWEET* genes studied (Fig. 7). At least one of these genes showed preferential expression during a specific stage of seed development. We observed that *AhSWEET24*, *AhSWEET46*, and *AhSWEET13/AhSWEET35* showed dramatic increase during 20–40 DAP across all four peanut varieties. The expression of *AhSWEET20*/*AhSWEET39* continuously increased from 20 to 60 DAP in ZH24. Moreover, the expression of *AhSWEET3*/*AhSWEET26* initially increased during 20–30 DAP, followed by a decrease during 40–60 DAP. *AhSWEET17* and *AhSWEET37* exhibited predominantly expression during 20–30 DAP, followed by a sharp decrease from 40 to 60 DAP. Meanwhile, *AhSWEET21/AhSWEET40* transcripts consistently maintained high levels of accumulation throughout all the five stages of seed development.

Most of the homologous gene pairs, such as *AhSWEET24* and *AhSWEET46*, showed similar expression changes. However, the homologous gene pair *AhSWEET23* and *AhSWEET44* demonstrated significantly different expression patterns across the four varieties. Specifically, compared to the other three varieties, the expression of *AhSWEET23* was primarily high at 20 DAP and 30 DAP in the SY131 variety. These results suggested that the twelve *AhSWEET* genes were likely to have some strong effects on the process of peanut seed development and might be players accounting for better quality in peanuts.

## Discussion

SWEET proteins are proposed to serve as essential sugar transporters in plants and play critical roles in regulating plant development and responding to various stresses [32]. Although SWEET proteins in *Arabidopsis* and rice have been extensively studied, the identification and functional characterization of SWEET proteins in other plants, including peanuts, are still limited. In this study, our focus lies in the characterization and evolution of the *SWEET* gene family and the identification of their potential functions in peanut seed development. A total of 94 *SWEET* genes were identified across three peanut species, including a cultivated peanut *A. hypogaea* (AABB genome) and its two wild progenitors, *A. duranensis* (AA genome) and *A. ipaensis* (BB genome). By comparison, the number of *SWEET* genes in *A. hypogea* (47) was equivalent to the combined total of *SWEET* genes in the two wild peanut species, *A. duranensis* (23) and *A. ipaensis* (24). Particularly noteworthy is the orthologous gene group analysis of *SWEETs* in the three peanut species, which showed that 86.96% of *AdSWEETs* and 83.33% of *AiSWEETs* were conserved in *A. hypogea*. Additionally, gene homology and phylogenetic analyses unveiled extensive homology among *SWEET* genes between diploid and tetraploid peanuts, consistent with previous reports demonstrating that *A. hypogaea* is derived from an initial hybridization event involving *A. duranensis* and *A. ipaensis* [20, 22, 24].

The common structural features of SWEET proteins, characterized by TMs, have previously undergone studies across multiple species, revealing both conservation and diversification [2, 6, 10, 33–35]. Through sequence analysis, we elucidated the TMs of peanut SWEET proteins and found that they exhibited a highly conserved structure with quantitative diversity ranged from four to eight. This suggested that these identified SWEET proteins are likely to possess SWEET protein functionality. Apart from the TMs structure analysis, high similarity was observed in gene structure, motifs, phylogenetic relationship, predicted subcellular localization, and predicted *cis*-elements within promoters between homologous *SWEET* gene pairs. These findings indicated that homologous genes deriving from the progenitors underwent few changes during evolution [36].

Whole genome duplication (WGD)/segment duplication and tandem duplication represent pivotal events in the expansion of gene families [37]. In *Brassica oleracea*, *SWEET* genes underwent seven WGD/segment duplication events, while in four cotton species, this number increased to 51 [2, 34]. Previous research has demonstrated that the genome of *A. hypogaea* has undergone a minimum of three WGD events during its evolution history [24]. Our results showed that 138 WGD/segment duplication events and 16 tandem duplication events coexist in peanut *SWEET* family, serving as the primary mechanisms accountable for the expansion in the total number of *SWEET* genes. This expansion mechanism in peanut *SWEET* family closely resembles what has been observed in cotton [34]. Importantly, WGD/segment duplication occurred at a significantly higher frequency than tandem duplication, indicating the pivotal role of the former in driving the expansion of peanut *SWEET* family. Altogether, the duplication events in peanut *SWEET* family contribute to the emergence of novel genes with distinct functions. Furthermore, our selective pressure analysis (Ka/Ks) identified purifying selection as the predominant driving force behind the evolution of peanut *SWEET* genes, which may play a crucial role in maintaining ancestral their biological functions.

Previous studies have provided evidence indicating that some *SWEET* genes are involved in plant seed development, with speculation that their function is to facilitate sugar transport across cellular membranes. For instance, *AtSWEET11*, *AtSWEET12*, and *AtSWEET15*, which are sucrose transporters, have been implicated in the regulation of seed coat and endosperm development [15]. In maize, the mutant of *ZmSWEET4c* led to a reduction in hexose transport at the basal endosperm transfer layer during seed filling [38]. In rice, *OsSWEET11* and *OsSWEET15*, which were highly and specifically expressed in caryopses, influenced the accumulation of starch in the pericarp [31]. Additionally, *GmSWEET10a*, a gene associated with soybean domestication, improved seed size and oil content by facilitating the transport of sucrose and hexose [39]. Inspired by these research progress, we conducted an analysis of peanut transcriptome data, which showed that twelve *AhSWEETs* (*AhSWEET13*, *17*, *20*, *21*, *23*, *24*, *26*, *37*, *39*, *40*, *44*, *46*) expressed to some extent in seeds. This indicated that these *AhSWEETs* may act a similar role as the homologous genes in *Arabidopsis*, rice, maize, and soybean [15, 31, 38, 39]. For example, *AhSWEET24* and *AhSWEET46* exhibited high expression levels from Pattee.6.seed to Pattee.10.seed. Their ortholog, *AtSWEET15*, was known to mediate seed development in *Arabidopsis* [15]. To gain a deeper insight into the function of the twelve *AhSWEETs* mentioned above in developing seeds, we conducted qRT-PCR analysis using four peanut varieties across five stages of seed development. The expression patterns of some genes in seeds (e.g. *AhSWEET23*, *AhSWEET44*, *AhSWEET3/26*) displayed some inconsistency at the same developmental stage across four varieties, potentially attributable to differences within the peanut varieties. We also found that *AhSWEET24*, *AhSWEET46*, and *AhSWEET13*/*AhSWEET35* exhibited the highest expression levels in developing seeds (from 30 DAP to 60 DAP) across all four varieties, indicated that they may be one of the major factors regulating the development of seeds in peanuts. These findings expand our understanding of the crucial role of *SWEET* genes in peanut seed development and the twelve *AhSWEET* genes may represent the factors contributing to enhanced seed quality in peanuts.

## Conclusions

In this study, a comprehensive and systematic investigation of the *SWEET* gene family across cultivated and wild peanuts were conducted. A total of ninety-four *SWEET* genes were identified in three peanut species. The examination of exon-intron structures, domain structures, and motifs reflected a combination of conservation and diversification among peanut *SWEET* genes. The evolutionary characteristics of the *SWEET* genes were elucidated through the comparison of phylogenetic relationships and the analysis of duplication events, including WGD/segmental duplication and tandem duplication. Ka/Ks analysis indicated that peanut *SWEET* genes had undergone strong purifying selection. Furthermore, the transcriptome data obtained from *A. hypogea* in various tissues and developmental stages highlighted that *AhSWEETs* were critical for the regulation of peanut growth and development. In particular, qRT-PCR results demonstrated that twelve *AhSWEET* genes were involved in peanut seed development process. Therefore, our findings provide valuable insights for future functional studies of *SWEET* genes and present potential candidate genes for improving the quality of peanut seeds.

## Materials and methods

### Identification of peanut *SWEET* genes

The genomic data of a cultivated peanut *A. hypogaea* cv. Tifrunner and two wild peanut species *A. duranensis* and *A. ipaensis* were obtained from PeanutBase (https://legacy.peanutbase.org/). Identification of SWEET proteins was performed using two tools: BLASTP search and HMMER (3.3.2 package). The protein sequences of *Arabidopsis* SWEETs from TAIR (https://www.arabidopsis.org/) were served as queries for BLASTP (e-value ≤ 1e-5) searches against the annotated peanut proteins and for identifying peanut SWEET homologs in the local peanut protein database. The conserved MtN3/saliva domain (PF03083) was then employed to identify the proteins through HMMER program (e-value ≤ 1e-5) [40]. By combining the resulting sequences obtained through the two methods, peanut SWEET proteins were identified. Orthologous genes among the three peanut species were identified using the OrthoFinder package with the default parameter “-f” [41].

The predicted molecular weights and theoretical isoelectric points were analyzed using ProtParam (https://web.expasy.org/protparam/). The TM helices (TMHs) were validated using SMART (http://smart.embl-heidelberg.de/). The ProtComp 9.0 (http://linux1.softberry.com/berry.phtml?topic=protcomppl&group=programs&subgroup=proloc) was used to predict the subcellular localization.

### Gene structure, protein motif and phylogenetic analysis

The GSDS2.0 (http://gsds.gao-lab.org/) was used to analyze the intron-exon organization. Conserved motifs were identified using MEME (https://meme-suite.org/meme/).

SWEET proteins sequences of *O. sativa*, *B. distachyon*, *S. italica*, *Z. mays*, *A. comosus*, *G. max* and *L. japonicus* were obtained from Phytozome V13 (https://phytozome-next.jgi.doe.gov/). The Neighbor-Joining phylogenetic tree was constructed using MEGA X software with a bootstrap of 1,000 replicates [42]. Then, the phylogenetic tree was visualized using iTOL v6 (https://itol.embl.de/).

### Chromosomal location, collinearity analysis, and Ka/Ks calculation

The chromosomal locations of peanut *SWEET* genes were obtained from GFF3 files and subsequently visualized using TBtools [43]. Based on BLASTP alignments (e-value < 1e-10), the collinearity within and between *A. hypogaea*, *A. duranensis* and *A. ipaensis* was established using MCScanX incorporated into TBtools [43]. The collinear relationships of *SWEET* genes were drawn using shinyCircos-V2.0 [44]. KaKs_Calculator (v 3.0) was used to calculate Ka/Ks values following sequence alignment of duplicated gene pairs using ParaAT2.0 [45, 46].

### Promoter *cis*-regulatory element prediction and transcriptome profile analysis

The 2,000 bp sequences upstream of the translational start sites of *SWEET* genes were extracted from the genomes of the three peanut species [20, 21]. The prediction of *cis*-regulatory elements was performed using PlantCARE (http://bioinformatics.psb.ugent.be/webtools/plantcare/html/).

The multi-tissue transcriptome data used in this study were obtained from Clevenger et al. [23]. Clean reads were extracted by filtering the raw data, followed by assembly into contigs and scaffolds using HISAT2 [47]. The expression levels of genes were calculated using StringTie2 (Version 2.1.4) [48].

### Plant materials, RNA isolation and qRT-PCR

In this study, four peanut varieties were utilized to investigate the relationship between the expression patterns of *SWEET* genes and sugar content during peanut seed development for qRT-PCR analysis. Zhonghua24 (var. *hypogaea*, ZH24) is a low-sucrose cultivar (sucrose content 2.54%) developed by Oil Crops Research Institute of the Chinese Academy of Agricultural Sciences (OCRI-CAAS), Wuhan, China in 2015. Nanyangbaipi (var. *hypogaea*, NYBP) is a landrace with low sucrose content (1.86%) collected by OCRI-CAAS. Jihuatian1 (var. *fastigiate*, JHT1) is a sweet cultivar with high sucrose content (7.35%) developed by Hebei Academy of Agriculture and Forestry Sciences, China in 2019. SY131 (var. *fastigiata*) is a high-sucrose breeding line (6.57%) developed by OCRI-CAAS. All four varieties were planted in the field at OCRI-CAAS.

Developing seeds at five different stages were harvested to conduct qRT-PCR analysis of twelve *AhSWEETs* across the four varieties. The five stages were as follow: 20 DAP (characterized by flat embryo), 30 DAP (featuring teardrop-shaped embryo), 40 DAP (exhibiting torpedo to round shaped embryo), 50 DAP (displaying round embryo), and 60 DAP (characterized by large and round embryo) [49].

Total RNA was extracted using TRIzol™ Reagent (Catalog Number 15,596,026, Invitrogen) following the user guide provided. Approximately 1 µg of total RNA was reverse-transcribed into cDNA with SuperScript™ IV (Catalog Number 18,090,010, Invitrogen). The peanut *actin* gene served as an internal control to normalize the expression levels [49]. Three independent biological replicates were used for qRT-PCR analysis. The primer sequences used were detailed in Table S10.

### Vector construction and protoplast transformation

An 864-bp coding sequence of *AhSWEET24* without the stop codon (the sense primer with *Spe* I restriction site: 5’-CGGACTAGTATGACGACCAATAATCATCCCA-3’ and the antisense primer with *Bam*H I restriction site: 5’-CGCGGATCCTCTAAGATGTGTTAGGTTGGAT-3’) was amplified from 40 DAP seeds of ZH24, and then was fused with the GFP reporter gene in pAN580 vector. As a control, the empty pAN580 vector was utilized. The 35 S::BnaA.FAE1-RFP vector is an endoplasmic reticulum (ER) marker [49]. The isolation and transformation of protoplasts were conducted using the *Arabidopsis* Protoplast Preparation and Transformation Kit (PPT101, Coolaber, Beijing, China). The 15-day-old *Arabidopsis* seedlings were used to isolate the protoplast cells. The leaves (~ 1 g) were lysed in a 10 mL solution containing cellulase and macerozyme for 4 h at 28 ℃. Subsequently, the protoplast solution containing plasmid DNA (~ 10 µg) was employed for PEG-mediated transformation. The 35 S::AhSWEET24-GFP and 35 S::BnaA.FAE1-RFP vectors were co-transfected into *Arabidopsis* protoplasts. Following an incubation for 10 h, the fluorescent proteins were detected using an Olympus FV10-ASW confocal microscope.

### Electronic supplementary material

Below is the link to the electronic supplementary material.

Supplementary Material 1

Supplementary Material 2

Supplementary Material 3

Supplementary Material 4

Supplementary Material 5

Supplementary Material 6

Supplementary Material 7

Supplementary Material 8

Supplementary Material 9

Supplementary Material 10

Supplementary Material 11

Supplementary Material 12

Supplementary Material 13

## Data Availability

All data used in this study are included in this article and additional files. The Genome sequence and annotation datasets are available in: PeanutBase (https://legacy.peanutbase.org/), TAIR (https://www.arabidopsis.org/) and Phytozome V13 (https://phytozome-next.jgi.doe.gov/). All the genes used in this study for phylogeny and subsequent analysis are mentioned in Table S1 and Table S4. Transcriptome data used for gene expression analysis are mentioned in Table S9.

## Abbreviations

SUTs: Sucrose transporters
MSTs: Monosaccharide transporters
SWEET: Sugar will eventually be exported transporter
TMs: Transmembrane helixes
THBs: Triple-helix bundles
TMHs: TM helices
DAP: Days after pollination
NYBP: Nanyangbaipi
ZH24: Zhonghua24
JHT1: Jihuatian1
ER: Endoplasmic reticulum
WGD: Whole genome duplication
Ka: Nonsynonymous substitution rate
Ks: Synonymous substitution rate
