# Supplementary figures and images for: Genome-wide identification of SWEET genes reveals their roles during seed development in peanuts
Source: BMC Genomics. 2024 Mar 7;25:259. doi: 10.1186/s12864-024-10173-w (PMC10921654; doi:10.1186/s12864-024-10173-w)

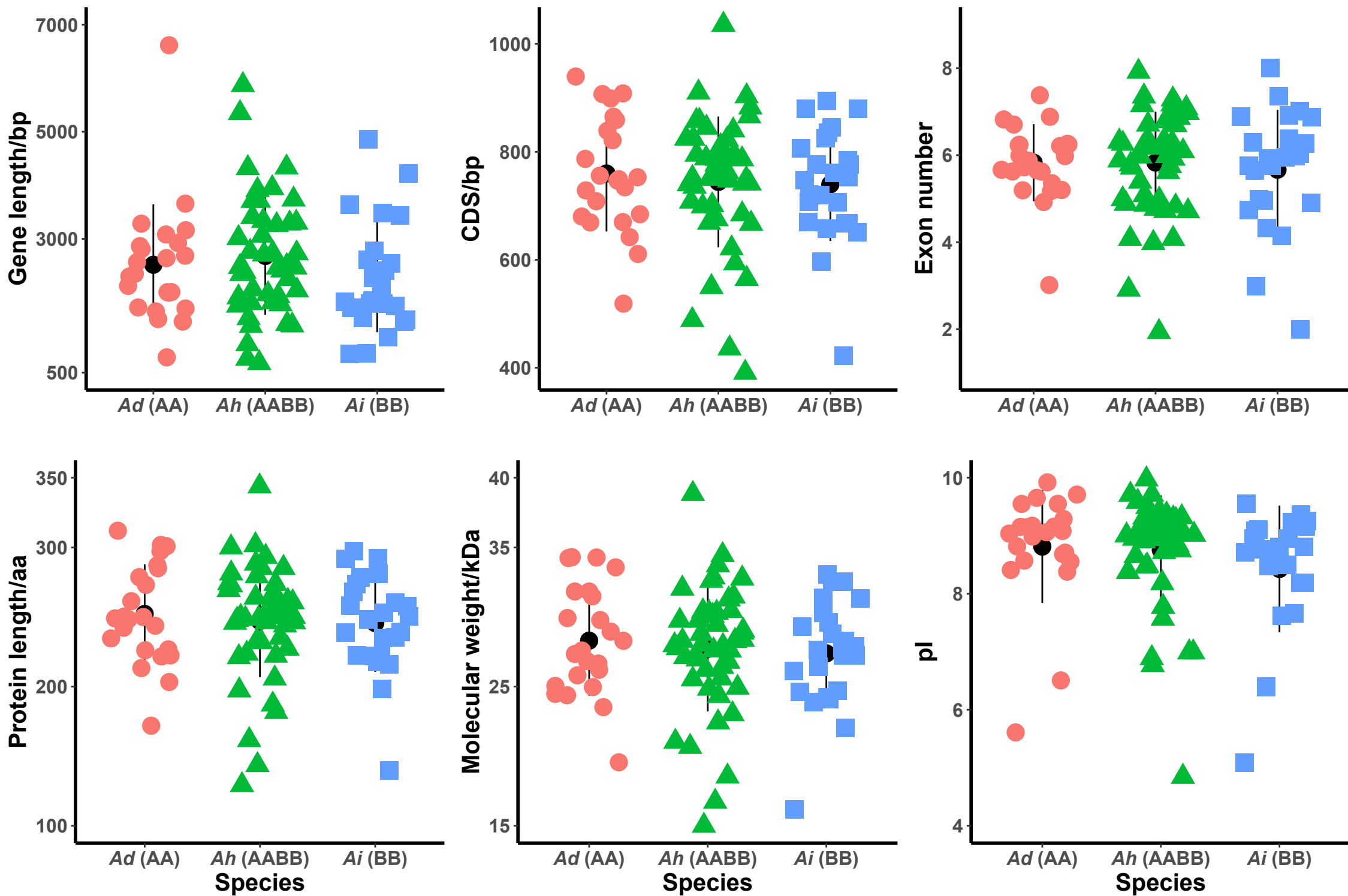

**Fig. S1** Characteristics of the *SWEET* genes in three peanut species.

Supplement: Supplementary file 11 — Supplementary Material 11 [file 12864_2024_10173_MOESM11_ESM.pdf]
