# Supplementary material for: Genome-wide identification of SWEET genes reveals their roles during seed development in peanuts
Source: BMC Genomics. 2024 Mar 7;25:259. doi: 10.1186/s12864-024-10173-w (PMC10921654; doi:10.1186/s12864-024-10173-w)
Supplement: Supplementary file 12 — Supplementary Material 12 [file 12864_2024_10173_MOESM12_ESM.pdf]

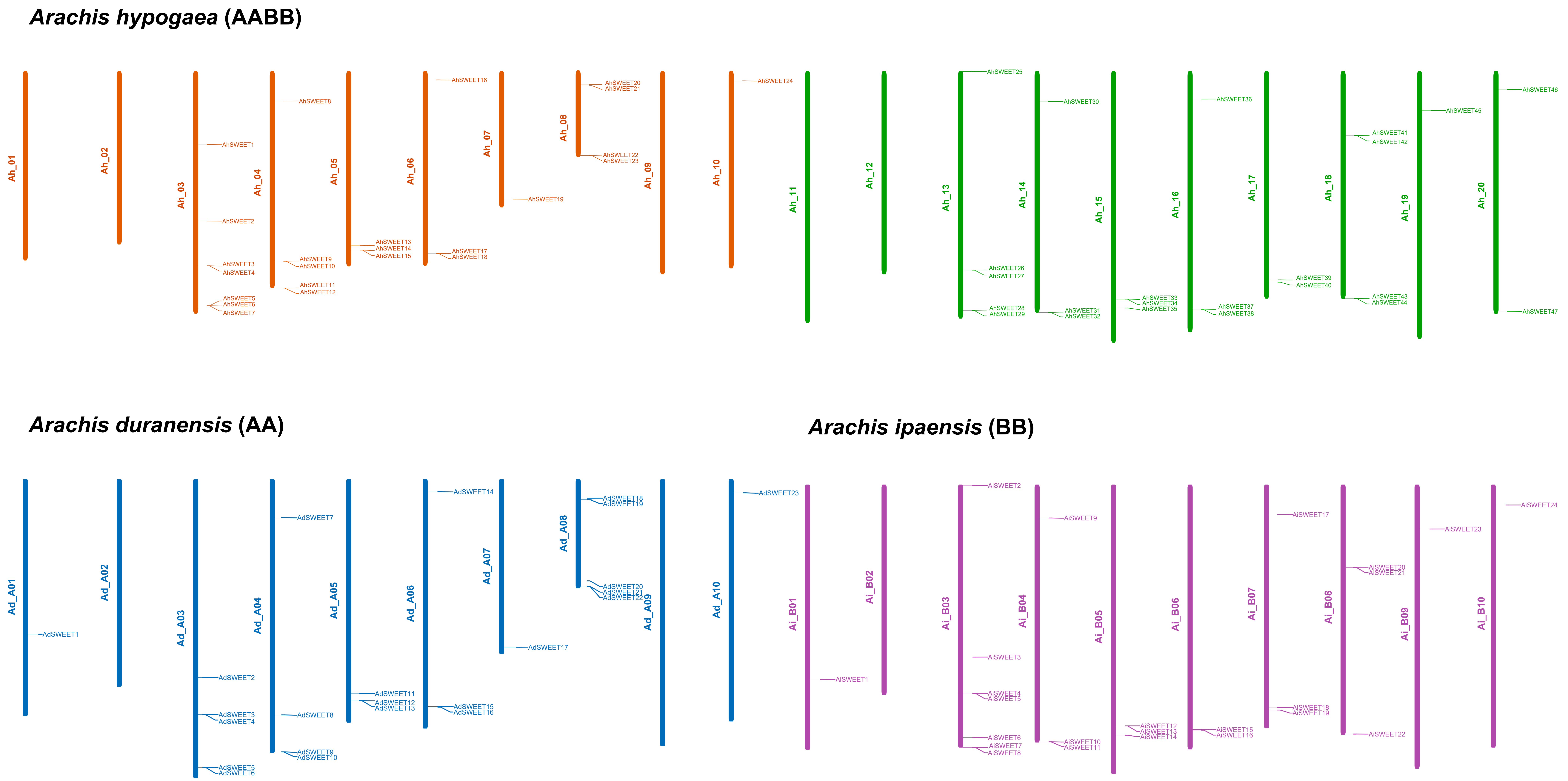

Fig. S2 Distributions of the *SWEET* genes in three peanut genomes.
