# Supplementary material for: Genome-wide identification of SWEET genes reveals their roles during seed development in peanuts
Source: BMC Genomics. 2024 Mar 7;25:259. doi: 10.1186/s12864-024-10173-w (PMC10921654; doi:10.1186/s12864-024-10173-w)
Supplement: Supplementary file 13 — Supplementary Material 13 [file 12864_2024_10173_MOESM13_ESM.pdf]

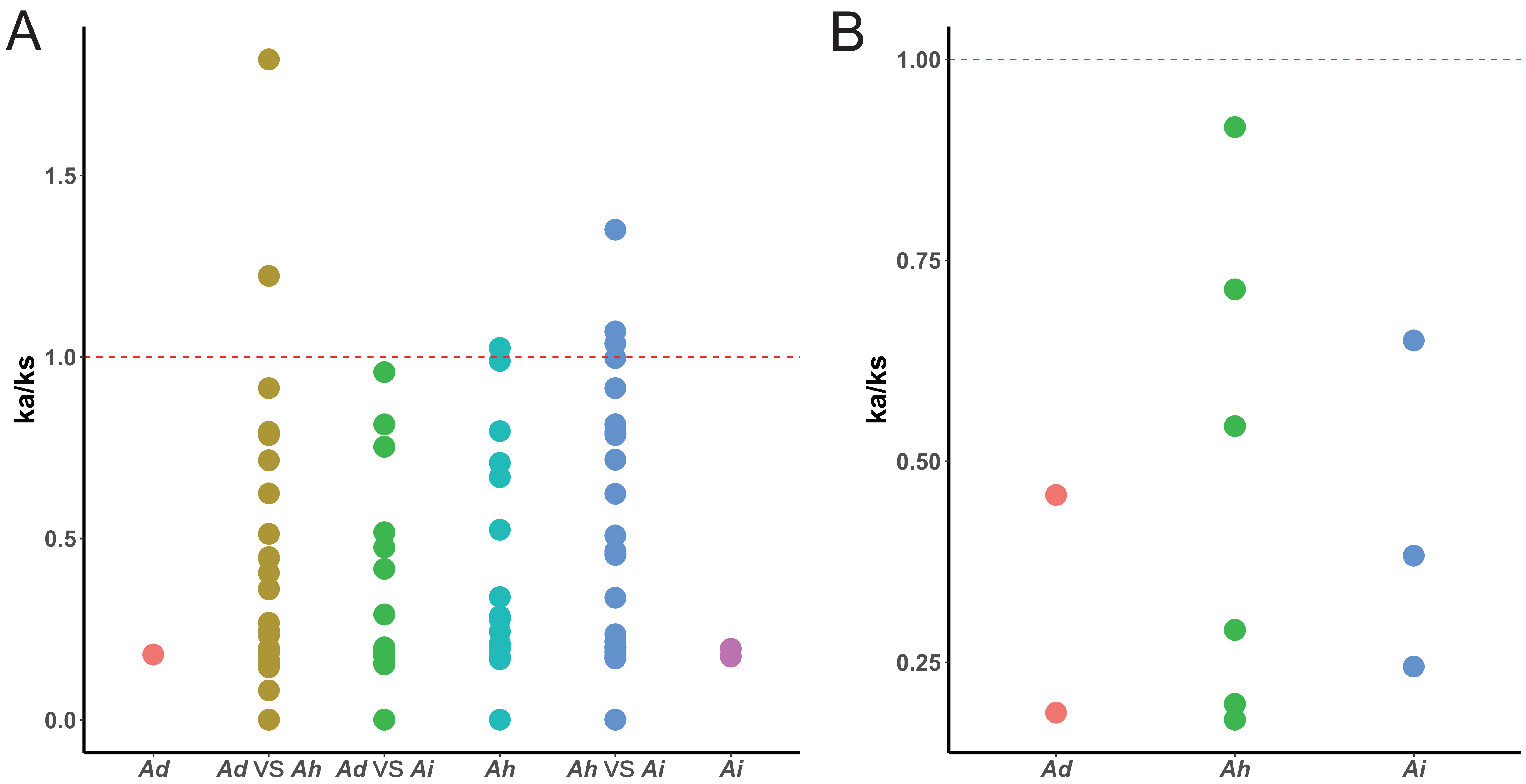

Fig. S3 Ka/Ks ratios of peanut *SWEET* genes.

(A) Ka/Ks ratios of collinear gene pairs. (B) Ka/Ks ratios of tandem duplicated genes.
